# Alarming Trend in Under-Five Indian Children’s Exposure to Indoor Tobacco Smoke

**DOI:** 10.7759/cureus.37571

**Published:** 2023-04-14

**Authors:** Bhupendra K Verma, Mamta Verma, Mukul Mondal, Dharmendra K Dubey, Dilip C Nath, Vivek Verma

**Affiliations:** 1 Law, Bimal Chandra College of Law, Murshidabad, IND; 2 Law, Kazi Nazrul University, Asansol, IND; 3 Biostatistics, School of Allied Health Sciences (SAHS) Sharda Hospital, Sharda University, Greater Noida, IND; 4 Applied and Pure Sciences, School of Applied and Pure Sciences, Royal Global University, Guwahati, IND; 5 Statistics, Assam University, Silchar, IND

**Keywords:** exposed to tobacco smoke, tobacco smoke, indian constitution, logistic regression, passive smoking

## Abstract

Background and aim: Children who are exposed to tobacco smoke (ETS) are at risk for a variety of health issues. There are enough legislative provisions in Indian law to safeguard children from ETS in outdoor settings, but no such specific rules exist to shield them from exposure indoors. This study aimed to examine the trend in under-five children's exposure to indoor tobacco smoke over the course of a decade (from 2005 to 2016) in India.

Materials and methods: Data from the National Family and Health Survey (NFHS) for the years 2005-2006 (NFHS-3) and 2015-2016 (NFHS-4) on under-five children have been taken into consideration in cross-sectional analyses of the Demographic and Health Survey on India. Based on different sociodemographic factors, the propensity of indoor ETS among Indian children has been estimated and compared using both bivariate and multivariate logistic regression models.

Results: The prevalence of indoor ETS among Indian children under the age of five has greatly risen over the past decade, rising from 4.12% to 52.70%. According to the findings, there has been a noticeable increase in every group of kids, regardless of their age, place of residence, geographic location, socioeconomic status, and literacy level of their mothers.

Conclusion: In India, the incidence of indoor ETS among children under five has risen by 13 times in the last 10 years, endangering the country. As a result, the Indian government must prepare to take legislative action to safeguard children by passing laws that forbid smoking inside.

## Introduction

Tobacco use has led to fiscal and human tragedy on a global scale, killing eight million people annually from preventable causes [1,2]. The majority of deaths (85%) involve direct or primary tobacco use, with the remaining 15% occurring in nonsmokers who were subjected to secondhand smoke [2]. There is no safe degree of exposure to tobacco because it has negative physical and psychological effects. This is true of all tobacco products, whether they are used first or second. Significant negative health impacts of secondhand smoke exposure include cardiovascular [3], respiratory [4], renal [5], and physiological issues [6]. Children are more susceptible to the negative effects of cigarette smoke exposure than adults because of their immature immune systems [7]. Children who are exposed to tobacco smoke (ETS) have an increased chance of developing asthma, wheezing, respiratory infections, and sudden infant death syndrome (SIDS) [8-10]. It results in pregnancy problems, mortality, morbidity, and low birth weight in pregnant women [11-13].

India's overall prevalence of tobacco use is 10.38%, and smokeless tobacco use is 21.38%, according to the Global Adult Tobacco Survey (GATS) performed in 2016-2017. The Indian legislative system and Indian government have made decisions in every area, beginning with production, dissemination, and consumption levels, which are necessary to safeguard health and healthcare quality for both main and secondhand smokers [14,15]. The Indian government has started a campaign to raise awareness of the harmful effects of tobacco smoke, which has been found to be very effective [15-17].

In order to control exposure in the outdoor environment and restrict the accessibility of any tobacco-related products, the Indian legislative system has enacted various laws both at the state as well as country levels, such as the Bihar Prohibition of Smoking Act 1954 (updated in 2020), Orissa Prohibition of Smoking Act 1961, Delhi Prohibition of Smoking and Non-smokers Health Protection Act 1996, Himachal Pradesh Prohibition of Smoking and Non-smokers Health Protection Act 1997, Rajasthan Prohibition of Smoking and Non-smokers Health Protection Act 1999, Assam Prohibition of Smoking and Non-smokers Health Protection Act 1999, West Bengal Prohibition of Smoking and Spitting and Protection of Health of Non-smokers and Minors Act, 2001, Andhra Pradesh Prohibition of Smoking Health Protection Act 2002, Tamil Nadu Prohibition of Smoking and Spitting Act 2002, Assam Students and Juvenile Smoking Act 1923, Prohibition of Smoking in Public Places Rules, 2002, etc. Many states such as Uttar Pradesh, Madhya Pradesh, Jharkhand, Chhattisgarh, Uttarakhand, and Maharashtra, have been following the guidelines of country laws of WHO Framework Convention on Tobacco Control (WHO FCTC) 2002, Cigarettes and Other Tobacco Products Act (COTPA) 2003, and WHO Framework Convention on Tobacco Control, 2015 (WHO FCPC).

ETS can take place indoors, outdoors, or both. The amount of exposure children encounter typically varies with their age, such as when they are exposed to their mothers as fetuses [18,19]. When a child is older than three years, both indoor and outdoor environments determine the amount of exposure [20,21]. From the neonatal to infant period, this is primarily dependent on the indoor environment [22,23].

Legislative safeguards in the open air and close to smoking places have received more attention in order to protect children [24], who are more susceptible to suffering negative effects from exposure to tobacco smoke. The purpose of this study was to compare the trend (2005-2016) of indoor tobacco smoke exposure experienced by children under the age of five in India and to map the associations between regional and socioeconomic characteristics with it in order to determine whether the current Indian legislative system on protection from ETS is effective in the indoor environment as well.

## Materials and methods

Secondary data from the Demographic and Health Survey (DHS) (https://dhsprogram.com/data/), which offers a nationally representative database containing data on the population, including information on the health, anthropometry, and nutritional status of chosen people, was used to perform cross-sectional studies. All living children who are five years old or younger are included in the study. Children older than five years who are deceased are excluded from the study. The National Family and Health Survey (NFHS) for the years 2005-2006 (NFHS-3) and 2015-2016 (NFHS-4) on Indian children under five years of age have been taken into consideration in the current research.

Outcome variable

The ETS experienced by the child under-five years of age in the indoor environment depends on whether any of the family members is a smoker or any outsider tobacco smoke inside the house. Under both of the situations where a child gets in secondhand smoke in an indoor environment has been considered as exposed to tobacco smoke, and in the notation of dichotomous variable denoted as "1," if not exposed then "0."

Study parameters

Based on different demographic characterizations, it has been studied how to understand the behavioral shift in ETS among Indian children in an indoor setting. The decadal shift in the sociodemographic distribution of exposure over the periods (2005-06 and 2015-16) has been compared, presuming that the current legislative enactments are capable of protecting children from exposure.

Gender and age of the child, residence, region, religion, caste, wealth index, gender of the head of household (HH), mother's age group, education, and profession, and father's education and occupation are some of the demographic factors that have been taken into account. The mother provided the child's age, which is divided into the following five distinct subgroups: 1, 1-2, 2-3, 3-4, and 4-5 years. Depending on their location, geographic zones have been categorized as Central, Eastern, North-Eastern, Northern, Southern, and Western. Hinduism, Islam, Christianity, and other religions are divided into these four categories. There are two categories of residence - urban and country. Based on their wealth index, the family's socioeconomic standing has been divided into the following categories: poorest, poorer, middle-class, richer, and richest. Mothers' ages were voluntarily recorded, and divided into seven distinct subgroups, including 15-19, 20-24,..., and 45-49. There are four categories for a parent's educational background - no formal schooling, primary, secondary, and higher. Five groups have been used to categorize parent occupations - not working (housewife/househusband), professional, agricultural, services, and skilled and unskilled manual-related duties. Two weeks previous to the survey, mothers were questioned about whether their kids had symptoms of acute respiratory infections (ARI). ARI is described as a chest-related cough that is followed by short, rapid, or challenging breathing. Information about the birth weight (in grams) has been classified into three groups viz., <2500 g, ≥2500 g, and not weighed at birth. To estimate and contrast the propensity of indoor ETS and its associated factors among Indian children over the past 10 years, both bivariate and multivariate logistic regression models were used. For all analytical work, SAS University Edition software has been used.

## Results

Table 1 presents the sociodemographic variation in the prevalence of exposure to indoor tobacco smoke experienced by under-five Indian children in a decade during 2005-06 to 2015-16, based on NFHS data. A substantial increase from 4.1% to 52.7% has been observed in the prevalence of exposure to indoor tobacco smoke. Based on the sociodemographic characteristics of children and geographical characteristics, obtained results have shown that in a decade significant increment in the prevalence of exposure to indoor tobacco smoke among male children (NFHS-3: 50.09%; NFHS-4: 52.1%), children below two years of age (NFHS-3: 32.72%; NFHS-4: 38.75%), urban residents (NFHS-3: 15.1%; NFHS-4: 25.87%), in all geographical regions (NFHS-3: 65.71%; NFHS-4: 76.34%) except Eastern, all religious groups except Christian, among the middle and above economical classes (NFHS-3: 25.74%; NFHS-4: 50%). At the levels of family composition a decade-significant increment in the prevalence of exposure to indoor tobacco smoke among children has been observed among families having mothers who are in the age group of 20-29 years (NFHS-3: 52.54%; NFHS-4: 71%), literate (NFHS-3: 27.74%; NFHS-4: 66.73%), are either not working (housewife) or engaged in professionals jobs (NFHS-3: 48.63%; NFHS-4: 78.47%), fathers having secondary or higher educational qualifications (NFHS-3: 32.06%; NFHS-4: 64.76%), and are either not working or engaged in professionals or services related jobs (NFHS-3: 17.7%; NFHS-4: 33.52%).

**Table 1:** Sociodemographic distribution of change in prevalence of exposure to indoor tobacco smoke among under-five Indian children from 2005 to 2016.

Variable		National family health survey-3 (2005-06) (4.1%)		National family health survey-4 (2015-16) (52.7%)		p-Value
		Total (n=52,868)	Exposed (n=2,178)	Total (n=238,930)	Exposed (n=125,912)	
Child						
Sex of child	Male	27,626	1,091 (50.09)	124,487	65,598 (52.1)	<0.0001
Child's age group	<1 year	10,403	364 (16.7)	46,103	24,012 (19.07)	<0.0001
	1-2 years	10,419	349 (16.02)	47,837	24,777 (19.68)	
	2-3 years	10,383	439 (20.16)	47,394	24,838 (19.73)	
	3-4 years	10,829	469 (21.54)	49,827	26,659 (21.17)	
	4-5 years	10,835	557 (25.58)	47,769	25,625 (20.35)	
Demography						
Country	India	52,868	2,178 (4.12)	238,930	125,912 (52.70)	<0.0001
Residence	Urban	13,665	329 (15.1)	67,984	32,569 (25.87)	<0.0001
Region	Central	15,354	685 (31.45)	63,410	40,608 (32.25)	<0.0001
	Eastern	13,322	747 (34.29)	60,758	29,788 (23.66)	
	North-Eastern	1,998	190 (8.73)	8,440	4,560 (3.62)	
	Northern	6,915	278 (12.78)	31,602	20,155 (16.01)	
	Southern	12,761	187 (8.59)	43,840	19,477 (15.47)	
	Western	2,518	91 (4.16)	30,880	11,325 (8.99)	
Religion	Hindu	41,284	1,609 (73.86)	187,790	99,268 (78.84)	<0.0001
	Muslim	9,085	476 (21.86)	39,558	20,680 (16.42)	
	Christian	1,058	58 (2.66)	4,970	2,794 (2.22)	
	Other	1,441	35 (1.62)	6,613	3,170 (2.52)	
Wealth quintile	Poorest	13,200	1,051 (48.25)	59,384	33,847 (26.88)	<0.0001
	Poorer	11,671	567 (26.01)	52,142	29,339 (23.3)	
	Middle	10,492	306 (14.05)	47,498	25,078 (19.92)	
	Richer	9,684	196 (8.98)	43,911	21,465 (17.05)	
	Richest	7,821	59 (2.71)	35,995	16,183 (12.85)	
Family						
Sex of head of household	Male	46,949	1,900 (87.21)	20,9673	111,582 (88.62)	0.0395
Mother's age group	15-19	3419	77 (3.55)	6,710	3,437 (2.73)	<0.0001
	20-24	18,660	479 (22)	76,740	40,247 (31.96)	
	25-29	17,746	665 (30.54)	93,464	49,035 (38.94)	
	30-34	8,529	499 (22.92)	41,998	22,005 (17.48)	
	35-39	3,348	294 (13.51)	15,012	8,270 (6.57)	
	40-44	947	135 (6.18)	3,921	2,261 (1.8)	
	45-49	220	28 (1.3)	1,085	658 (0.52)	
Mother education	No education	25,960	1,574 (72.26)	70,615	41,894 (33.27)	<0.0001
	Primary	7,401	317 (14.57)	33,232	19,155 (15.21)	
	Secondary	16,749	270 (12.38)	109,446	53,901 (42.81)	
	Higher	2,756	17 (0.79)	25,637	10,963 (8.71)	
Father education	No education	15,138	1,064 (49.13)	7,107	4,330 (19.19)	<0.0001
	Primary	7,812	391 (18.06)	5,957	3,571 (15.83)	
	Secondary	23,961	629 (29.07)	22,423	11,930 (52.88)	
	Higher	5,353	65 (2.99)	5,827	2,681 (11.88)	
	Don't know	484	16 (0.75)	109	47 (0.21)	
Mother's occupation	Not working	33,363	1,039 (47.73)	32,673	17,365 (76.92)	<0.0001
	Professional	798	20 (0.9)	762	349 (1.55)	
	Agricultural	13,308	839 (38.51)	4,890	3,065 (13.58)	
	Services	1,574	70 (3.22)	1,303	660 (2.93)	
	Skilled and unskilled manual	3,814	210 (9.64)	1,819	1,135 (5.03)	
Father's occupation	Did not work	547	26 (1.19)	2,266	1,208 (5.36)	<0.0001
	Professional	2,939	46 (2.13)	3,112	1,397 (6.19)	
	Agricultural	16,233	793 (36.45)	12,216	6,967 (30.88)	
	Services	11,044	313 (14.38)	9,850	4,957 (21.97)	
	Skilled and unskilled manual	21,989	997 (45.86)	13,979	8,030 (35.59)	
ARI in child	Yes	4,952	257 (11.79)	13,332	7,435 (5.90)	<0.0001
Birth weight (in grams)	<2,500	3,961	102 (4.72)	33,357	17,282 (13.73)	<0.0001
	>2,500	14,723	302 (13.88)	155,636	78,886 (62.65)	
	Not weighed	34,184	1,773 (81.40)	49,937	29,745 (23.62)	

The decadal change in the determinants of the prevalence of exposure to indoor tobacco smoke among determinants of prevalence has been presented in Tables 2, 3 by using both bivariate and multivariate logistic regression analysis, respectively. The unadjusted analysis of bivariate logistic regression based on NFHS-3 (2005-2006) revealed that child gender, age groups 2-5 years, geographical regions Central, Eastern, and North-Eastern, all religions (highest among Muslims), all economical classes (highest among poorest followed by poorer), mothers of age-groups 25-49 years (highest among 45-49 years), both literate and illiterate mothers (highest among illiterates), working mothers (highest among agricultures), and both literate and illiterate father (highest non-educated) have significant effect on the prevalence of exposure to indoor tobacco smoke among children (Table 2). After a decade (based on NFHS-4 {2015-16}) the results revealed that children of age groups 3-5 years, all geographical regions, all religions (highest among Christian), all economical classes (highest among poorest followed by poorer), families having male head of household (HH), mothers of age-groups 20-49 years (highest among 45-49 years), both literate and illiterate mothers (highest among illiterates), both working and housewife mothers (highest among agricultures), both literate and illiterate father (highest non-educated), father working as agricultural labor or skilled and unskilled manual, have significant effect on the prevalence of exposure to indoor tobacco smoke among children.

**Table 2:** Unadjusted odds ratio of exposure to indoor tobacco smoke among Indian children under five years of age from 2005 to 2016.

Variable		Odds ratio unadjusted (95%CI)	
		National family health survey-3 (2005-2006)	National family health survey-4 (2015-2016)
Sex of child (reference: male)	Female	1.09 (1.00-1.19)	1.00 (0.98-1.02)
Child's age group (reference: <1 year)	1-2 years	0.96 (0.82-1.11)	0.99 (0.96-1.01)
	2-3 years	1.22 (1.06-1.40)	1.01 (0.99-1.04)
	3-4 years	1.25 (1.09-1.44)	1.06 (1.03-1.09)
	4-5 years	1.50 (1.31-1.71)	1.06 (1.04-1.09)
Residence (reference: rural)	Urban	0.50 (0.44-0.56)	0.76 (0.75-0.78)
Region (reference: Western)	Central	1.25 (1.00-1.57)	3.08 (2.99-3.16)
	Eastern	1.59 (1.27-1.99)	1.66 (1.61-1.71)
	North-Eastern	2.82 (2.18-3.65)	2.03 (1.93-2.13)
	Northern	1.12 (0.88-1.43)	3.04 (2.94-3.14)
	Southern	0.40 (0.31-0.51)	1.38 (1.34-1.42)
Religion (reference: other)	Hindu	1.61 (1.15-2.26)	1.22 (1.16-1.28)
	Muslim	2.20 (1.56-3.11)	1.19 (1.13-1.25)
	Christian	2.30 (1.50-3.53)	1.39 (1.29-1.50)
Wealth quintile (reference: richest)	Poorest	11.38 (8.74-14.82)	1.62 (1.58-1.67)
	Poorer	6.71 (5.13-8.79)	1.58 (1.53-1.62)
	Middle	3.95 (2.99-5.23)	1.37 (1.33-1.41)
	Richer	2.71 (2.02-3.64)	1.17 (1.14-1.20)
Sex of head of household (reference: female)	Male	0.85 (0.75-0.97)	1.19 (1.16-1.21)
Mother's age group (reference: 15-19 years)	20-24	1.14 (0.89-1.45)	1.05 (1.00-1.10)
	25-29	1.69 (1.33-2.14)	1.05 (1.00-1.10)
	30-34	2.69 (2.11-3.43)	1.05 (1.00-1.10)
	35-39	4.17 (3.23-5.39)	1.17 (1.10-1.24)
	40-44	7.17 (5.37-9.59)	1.30 (1.20-1.40)
	45-49	6.39 (4.06-10.07)	1.47 (1.29-1.67)
Mother education (reference: higher)	No education	10.22 (6.35-16.45)	1.95 (1.90-2.01)
	Primary	7.10 (4.36-11.54)	1.82 (1.76-1.88)
	Secondary	2.59 (1.59-4.22)	1.30 (1.26-1.34)
Father education (reference: higher)	No education	6.21 (4.82-8.00)	1.95 (1.90-2.01)
	Primary	4.31 (3.31-5.62)	1.82 (1.76-1.88)
	Secondary	2.21 (1.71-2.86)	1.30 (1.26-1.34)
	Don't know	2.85 (1.64-4.95)	0.89 (0.61-1.30)
Mother's occupation (reference: professional)	Not working	1.28 (0.81-2.02)	1.30 (1.13-1.50)
	Agricultural	2.68 (1.70-4.22)	1.98 (1.70-2.31)
	Services	1.86 (1.12-3.09)	1.21 (1.01-1.45)
	Skilled and unskilled manual	2.32 (1.45-3.71)	1.96 (1.65-2.32)
Father's occupation (reference: did not work)	Professional	0.35 (0.22-0.55)	0.74 (0.69-0.80)
	Agricultural	1.11 (0.76-1.62)	1.21 (1.16-1.25)
	Services	0.63 (0.43-0.93)	0.92 (0.89-0.96)
	Skilled and unskilled manual	1.03 (0.71-1.49)	1.23 (1.19-1.27)
ARI in child (reference: no)	Yes	1.31 (1.15-1.50)	1.14 (1.10-1.18)
Birth weight (in grams) (reference: ≥2500)	<2500	1.27 (1.01-1.59)	1.05 (1.02-1.07)
	Not weighed	2.61 (2.31-2.95)	1.43 (1.40-1.46)

**Table 3:** Adjusted odds ratio of exposure to indoor tobacco smoke among Indian children under five years of age from 2005 to 2016.

Variable		Odds ratio adjusted (95% CI)	
		National Family Health Survey-3 (2005-06)	National Family Health Survey-4 (2015-16)
Sex of child (reference: male)	Female	0.98 (0.91-1.07)	1.01 (0.99-1.02)
Child's age group (reference: <1 year)	1-2 Years	0.98 (0.86-1.13)	1.03 (1.00-1.06)
	2-3 Years	1.14 (1.00-1.30)	1.03 (1.00-1.06)
	3-4 Years	1.11 (0.97-1.26)	1.06 (1.03-1.09)
	4-5 Years	1.14 (1.00-1.30)	1.07 (1.04-1.10)
Residence (reference: rural)	Urban	1.22 (1.10-1.36)	1.02 (1.00-1.05)
Region (reference: Western)	Central	0.87 (0.68-1.13)	2.66 (2.57-2.76)
	Eastern	1.35 (1.05-1.73)	1.24 (1.19-1.28)
	North-Eastern	4.72 (3.70-6.01)	2.39 (2.29-2.49)
	Northern	1.00 (0.77-1.28)	3.12 (3.01-3.24)
	Southern	0.36 (0.27-0.49)	1.13 (1.09-1.18)
Religion (reference: other)	Hindu	1.77 (1.43-2.18)	1.31 (1.25-1.36)
	Muslim	1.75 (1.39-2.19)	1.24 (1.19-1.30)
	Christian	1.69 (1.37-2.10)	2.67 (2.53-2.82)
Wealth quintile (reference: richest)	Poorest	3.61 (2.91-4.48)	1.30 (1.25-1.35)
	Poorer	2.26 (1.84-2.78)	1.34 (1.29-1.38)
	Middle	2.31 (1.91-2.79)	1.30 (1.25-1.34)
	Richer	1.83 (1.53-2.19)	1.19 (1.16-1.23)
Sex of head of household (reference: female)	Male	0.90 (0.79-1.02)	1.10 (1.07-1.13)
Mother's age group (reference: 15-19)	20-24	1.06 (0.86-1.32)	1.06 (1.00-1.11)
	25-29	1.26 (1.02-1.56)	1.04 (0.99-1.10)
	30-34	1.59 (1.27-1.98)	1.04 (0.98-1.10)
	35-39	1.75 (1.38-2.21)	1.07 (1.00-1.13)
	40-44	2.31 (1.75-3.06)	1.11 (1.03-1.20)
	45-49	1.69 (1.09-2.61)	1.07 (0.95-1.21)
Mother education (reference: higher)	No education	1.52 (1.15-2.01)	1.53 (1.48-1.59)
	Primary	1.52 (1.15-2.02)	1.48 (1.42-1.54)
	Secondary	1.33 (1.03-1.72)	1.27 (1.23-1.31)
Father education (reference: higher)	No education	1.01 (0.82-1.24)	0.98 (0.91-1.06)
	Primary	1.02 (0.82-1.26)	1.05 (0.97-1.14)
	Secondary	0.89 (0.74-1.07)	0.98 (0.92-1.05)
	Don't know	0.86 (0.55-1.35)	0.67 (0.47-0.97)
Mother's occupation (reference: professional)	Not working	0.60 (0.45-0.80)	0.85 (0.73-0.99)
	Agricultural	0.78 (0.58-1.05)	1.12 (0.95-1.32)
	Services	0.92 (0.67-1.26)	0.96 (0.80-1.15)
	Skilled and unskilled manual	0.64 (0.46-0.88)	1.06 (0.88-1.26)
Father's occupation (reference: did not work)	Professional	0.72 (0.52-1.01)	0.91 (0.83-1.00)
	Agricultural	0.78 (0.58-1.04)	0.99 (0.94-1.05)
	Services	0.72 (0.53-0.96)	0.97 (0.91-1.03)
	Skilled and unskilled manual	0.79 (0.60-1.06)	1.12 (1.06-1.19)

The adjusted analysis of multivariate logistic regression based on NFHS-3 (2005-2006) has revealed that the determinants having significant effect on the prevalence of exposure to indoor tobacco smoke among children are children of age groups 2-3 and 4-5 years, urban residents, geographical regions Eastern and North-Eastern, all religions (highest among Muslims), all economical classes (highest among poorest followed by poorer), and mothers of age-groups 25-49 years (highest among 45-49 years), and both literate and illiterate mothers (highest among illiterates). The multivariate logistic regression based on NFHS-4 (2015-2016) has revealed that the determinants having significant effect on the prevalence of exposure to indoor tobacco smoke among children are children of age groups 2-5 years, urban residents, all geographical regions, all religions (highest among Muslims), all economical classes (highest among poorest followed by poorer), families having male head of household (HH), mothers of age-groups 20-24 and 35-44 years (highest among 40-44 years), and both literate and illiterate mothers (highest among illiterates).

**Figure 1:**
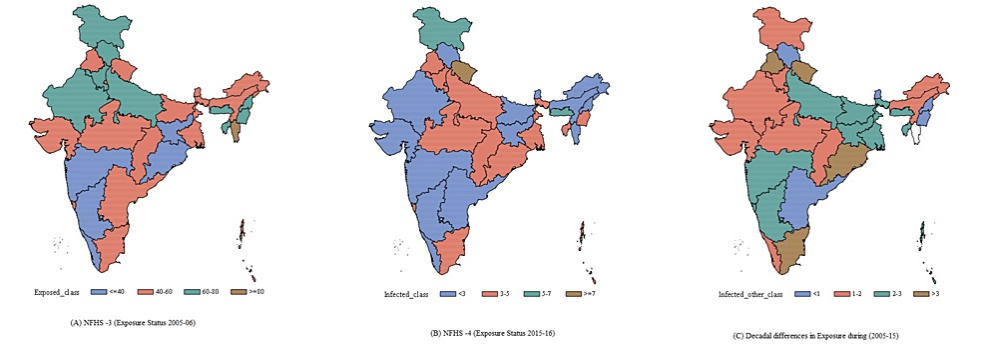
State-wise distribution of ETS during (A) 2005-2006, (B) 2015-2016, and (C) decadal differences in 2005-2015.

State-wise decadal changes in the prevalence of exposure to indoor tobacco smoke among children have been depicted in Figure 1. In a decade significant rise in the prevalence has been observed throughout all states and most states reported more than 20% increment.

## Discussion

Strict laws are needed to control the ETS and safeguard people from different health risks that can result in morbidity and mortality. The government is very worried about this problem because smoke exposure or inhalation has an impact on all forms of production and consumption. Children are severely impacted whenever they come into either direct or indirect touch with tobacco smoke. ETS has an effect on the infant from the time of conception until birth and even after. The kid may encounter this exposure in both indoor and outdoor settings. ETS can occur outdoors for a variety of reasons, but indoors it only happens because of family members or visitors who smoke inside. Taking into account the contributing factors, nations created their legal frameworks so that children's health could be safeguarded from exposure to cigarette smoke both indoors and out.

According to the results of the current research, there has been a significant rise in the prevalence of exposure to indoor tobacco smoke from 4.1% to 52.7%, which should worry both the government and policymakers. All regional areas and religious groups have seen an increase in the frequency of exposure to indoor tobacco smoke over the past 10 years. The prevalence of tobacco smoke exposure is notably high among children, regardless of the socioeconomic classes, age groups of the mothers (highest among 45-49 years), literacy of the mothers (highest among illiterates), employment of the mothers (highest among farmers), and literacy of the fathers (highest non-educated). Additionally, it was noted that when those heads of household were male, their prevalence increased decadally, whereas when they were female, they were discovered in decreasing order.

There are only a few indoor laws in India, including COTPA 2003, the Cigarettes and Other Tobacco Products (Prohibition of Advertisement and Regulation and Distribution) (COTP) Rules 2004, the Cable Television Networks Advertisement Act 1955, the Delhi Prohibition of Smoking and Non-smokers Health Protection Act 1996, and the West Bengal Prohibition of Smoking and Smog Act, 2001, which is made to protect non-smokers from ETS in an outdoor environment.

To restrict indoor ETS among children, some countries have enacted specific laws that prohibited tobacco smoking in indoor places, workplaces, and inside public transport, such as in England (The Health Act 2006), the United States of America (Family Smoking Prevention and Tobacco Control Act 2009), Australia (Smoke-Free Environment Act 2000, Smoke-Free Public Places Regulation 2005, and the Smoke-free Environment Regulation 2016), New South Wales (Smoke-Free Environment Act 2000), Ireland (The Public Health {Tobacco} Act 2002), Germany (The Protection of Young Protection Act, 2007), Bhutan (Tobacco Control Act of Bhutan 2010), Brazil (WHO Framework Convention on Tobacco Control {WHOFCTC}, 2006), and Spain (WHOFCTC in 2005).

The present study's limitations include the inability to link the effects of maternal and child exposure to other health parameters, such as the physiological, psychological, and cardiovascular systems, without knowledge of the quantity and duration of tobacco use indoors by family members or anyone else. In addition, more clinical research and pertinent data on mothers and children are needed in order to frame a policy to reduce associated risks and better understand the effect of ETS on child health.

## Conclusions

India saw major advancements in maternal care, vaccination coverage, newborn and child mortality rates control, literacy, health services, and other areas over the course of a decade. But since then, the majority of states have seen a marked increase in the frequency of indoor tobacco smoke. Over the past 10 years, the prevalence of indoor ETS among children under the age of five has risen by 13 times in India. The increased decadal shift in indoor ETS prevalence among Indian children under five years puts the nation in peril. The Indian Government must therefore follow suit in light of this dangerous situation and prepare legislative interventions to safeguard children and their childhood by enacting laws against indoor smoking. In other developed countries, strict laws have been passed outlawing tobacco use in indoor settings, such as workplaces and public transportation.
